# CONNECTED: leveraging digital twins and personal knowledge graphs in healthcare digitalization

**DOI:** 10.3389/fdgth.2023.1322428

**Published:** 2023-12-07

**Authors:** Antonella Carbonaro, Alberto Marfoglia, Filippo Nardini, Sabato Mellone

**Affiliations:** ^1^ Department of Computer Science and Engineering, Università di Bologna, Cesena, Italy; ^2^ Department of Industrial Engineering, Università di Bologna, Bologna, Italy; ^3^ Department of Electrical, Electronic, and Information Engineering “Guglielmo Marconi”, Università di Bologna, Cesena, Italy

**Keywords:** healthcare, digital twins, architectural framework, personal knowledge graphs, data integration

## Abstract

Healthcare has always been a strategic domain in which innovative technologies can be applied to increase the effectiveness of services and patient care quality. Recent advancements have been made in the adoption of Digital Twins (DTs) and Personal Knowledge Graphs (PKGs) in this field. Despite this, their introduction has been hindered by the complex nature of the context itself which leads to many challenges both technical and organizational. In this article, we reviewed the literature about these technologies and their integrations, identifying the most critical requirements for clinical platforms. These latter have been used to design CONNECTED (COmpreheNsive and staNdardized hEalth-Care plaTforms to collEct and harmonize clinical Data), a conceptual framework aimed at defining guidelines to overcome the crucial issues related to the development of healthcare applications. It is structured in a multi-layer shape, in which heterogeneous data sources are first integrated, then standardized, and finally used to realize general-purpose DTs of patients backed by PKGs and accessible through dedicated APIs. These DTs will be the foundation on which smart applications can be built.

## 1. Introduction

In the era of rapid technological advancements, digitalization has become a game-changer in healthcare, with the potential to improve patient care quality and safety (1). However, the successful realization of the digital revolution requires addressing several issues such as data fragmentation, lack of interoperability, and management of vast streams of real-time data (2). Modern technologies, such as Digital Twin (DT), emerged as a response to these challenges (3), but they are usually designed to have a vertical perspective which limits their capability to synergistically cooperate (4).

On the other hand, precision medicine has been a hot topic in recent years, improving the effectiveness and decreasing the side effects of clinical treatments (5). Personal Knowledge Graph (PKG) emerged as a promising solution to provide tailored services to its users (6), offering a comprehensive and patient-centric perspective on the individual’s health. Hence, there is an urgent need for architectural guidelines aimed at facilitating the development of an ecosystem of Digital Twins capable of communicating, which will act as a foundation for the implementation of healthcare applications.

In this perspective, we propose CONNECTED (COmpreheNsive and staNdardized hEalth-Care plaTforms to collEct and harmonize clinical Data), a conceptual multi-level framework aimed at integrating heterogeneous data sources using modern healthcare standards. The gathered information is fed to PKGs, which will support general-purpose patient DTs. Finally, custom applications can communicate with DTs through their APIs, realizing specific tasks, services, simulations, etc.

In the forthcoming sections, we will first review the existing literature on PKGs and DTs. Next, we will analyze current architectural approaches that incorporate these technologies, aiming to identify shared features. These highlighted characteristics will serve as the foundation for establishing the essential requirements that will guide the development of our framework.

## 2. Recent advancements in personal knowledge graph

In the existing literature, PKGs have been defined in multiple ways (6). According to Balog and Kenter (7), a PKG is characterized as a source of structured knowledge about entities and their relationships, where the entities and relationships are of personal importance to the user. Diverging from conventional knowledge graphs, a PKG is intentionally crafted to capture an individual’s personal knowledge and experiences, embodying a spiderweb-like layout. Thus, the primary aim is to facilitate the provision of services tailored specifically to its owner. In the current landscape, there has been significant interest in the application of PKGs, which have been employed in several healthcare applications.

Gyrard et al. propose a PKG framework aimed at developing personalized healthcare applications to effectively manage chronic disease (8). Rastogi and Zaki suggest that PKGs could be used to personalize recommendations from good platforms to encourage a healthy lifestyle (9). Ammar et al. present a mobile health digital intervention that combines both patients’ personal health data and contextual knowledge from various sources (10). The objective is to offer tailored recommendations for enhancing self-care behaviors among diabetic adults. Seneviratne et al. introduce a PKG that supports the generation of tailored dietary recommendations for Type 2 Diabetes self-management (11). To do so, reasonings are made over the PKG, that encodes clinical guidelines represented in OWL.

## 3. Digital twin in healthcare

DTs have found application in many healthcare sectors, such as oncology (12), cardiology (13), geriatrics (14), neurology (15), internal medicine (16), trauma management (17), pharmaceutics (18), orthopedics (19), surgery (20).

Croatti et al. discussed the application of agent-based DTs to digitalize the process of severe trauma management, realizing a continuous monitoring (17). A pre-hospital DT collects real-time information from the central unit, GPS systems, and smart rescuers’ equipment, registers the severity of the ongoing trauma decided by the rescuers, and automatically compiles emergency forms. Information is later sent to another DT aimed at managing the trauma process inside the hospital. Its life cycle evolves according to the macrosteps related to trauma and terminates when the patient is ready for hospitalization.

Björnsson et al. proposed to build DTs of patients, which can be computationally treated with thousands of drugs to find the optimal one, evaluating each one’s pros and cons (18).

Aubert et al. developed a virtual replica to evaluate stabilization method variants for tibial plateau fracture surgery at distinct stages of bone healing (19). It demonstrates how DTs can provide quantitative and high-value information for clinical decision-making.

## 4. Architectural frameworks for digital twins

In the domain of DTs, the establishment of robust architectural frameworks serves as a cornerstone for molding their functionalities and expanding their utility (21). This section delves into a selection of significant contributions that introduce architectural models designed to build and elevate DTs across different domains. After an analysis of each one, they will be summarized to highlight their main capabilities. Subsequently, these functionalities will provide the basis for developing our framework’s requirements.

Malakuti et al. provide an abstract four-layer architecture pattern to construct DTs in the industrial context (22). In their vision, the bottom layer consists of many heterogeneous information sources that feed data to the DTs through the Model layer, which aggregates information. The processed data is made accessible to applications through specialized DT APIs. Remarkably, this framework is intentionally engineered to be both extensible, accommodating a dynamic range of information sources, and flexible, enabling it to adeptly handle a variety of data types, making it a versatile foundation for DT development.

Sahlab et al. conceptualize a multi-layer KG-based architecture for the application of DTs in Cyber-Physical Systems, providing improved capabilities, such as internal linking and referencing, knowledge completion, error detection, collective reasoning, and semantic querying (23). The result is the Intelligent DT, an evolution of the DT, equipped with a module for model comprehension using artificial intelligence to continuously adapt and learn. They propose a modular solution in which the communication between physical and virtual assets is bidirectional. It also includes a reasoning engine, aimed at deducing knowledge from heterogeneous models and running predictions of future states of the system.

Ricci et al. discuss a vision in which healthcare, a complex domain, can be modeled as an open ecosystem of DTs, owned by different organizations, but designed to communicate and cooperate in the same scenarios (4). In this perspective, DTs are not bound to a specific application, but they are thought more of as services exposing their APIs, leading to a Digital Twin-as-a-service approach. They shift away from the traditional vertical representation of DT, embracing a layered architecture in which their capabilities can be horizontally composed. Within this vision, DTs serves as a stable foundation on top of which applications and smart algorithms can be built.

The work presented underlines features that address shared crucial needs of DT frameworks, from which it was possible to explicitly derive the requirements of CONNECTED:

• *Real-time data streaming*: The framework must incorporate ports to enable the real-time flow of data streams. Therefore, it necessitates a layer capable of gathering real-time data from IoT devices, medical devices, smartphones, organizations, etc.
• *Heterogeneous sources integration*: It must be possible to collect data from diverse sources and feed them to the relevant DTs. To do that, the gathered information must be harmonized to a common domain model. Especially in the healthcare context, standards might facilitate this process, also improving interoperability across organizations.
• *Symbolic reasoning*: As Knowledge Graphs can effectively describe and manage healthcare information, by employing reasoning algorithms, they facilitate the derivation of insights regarding a patient’s status and prognostications. Furthermore, the stored knowledge can be accessed through intuitive semantic queries, in an explorative search fashion.
• *General-purpose Digital Twin*: DTs should act as a foundation on top of which smart applications can be developed. Hence, they do not need to be aware of specific program details. In this regard, DTs can be represented by services exposing proper APIs, enabling effective communication with external software.

## 5. CONNECTED: a framework for general-purpose digital twins

**Figure 1:**
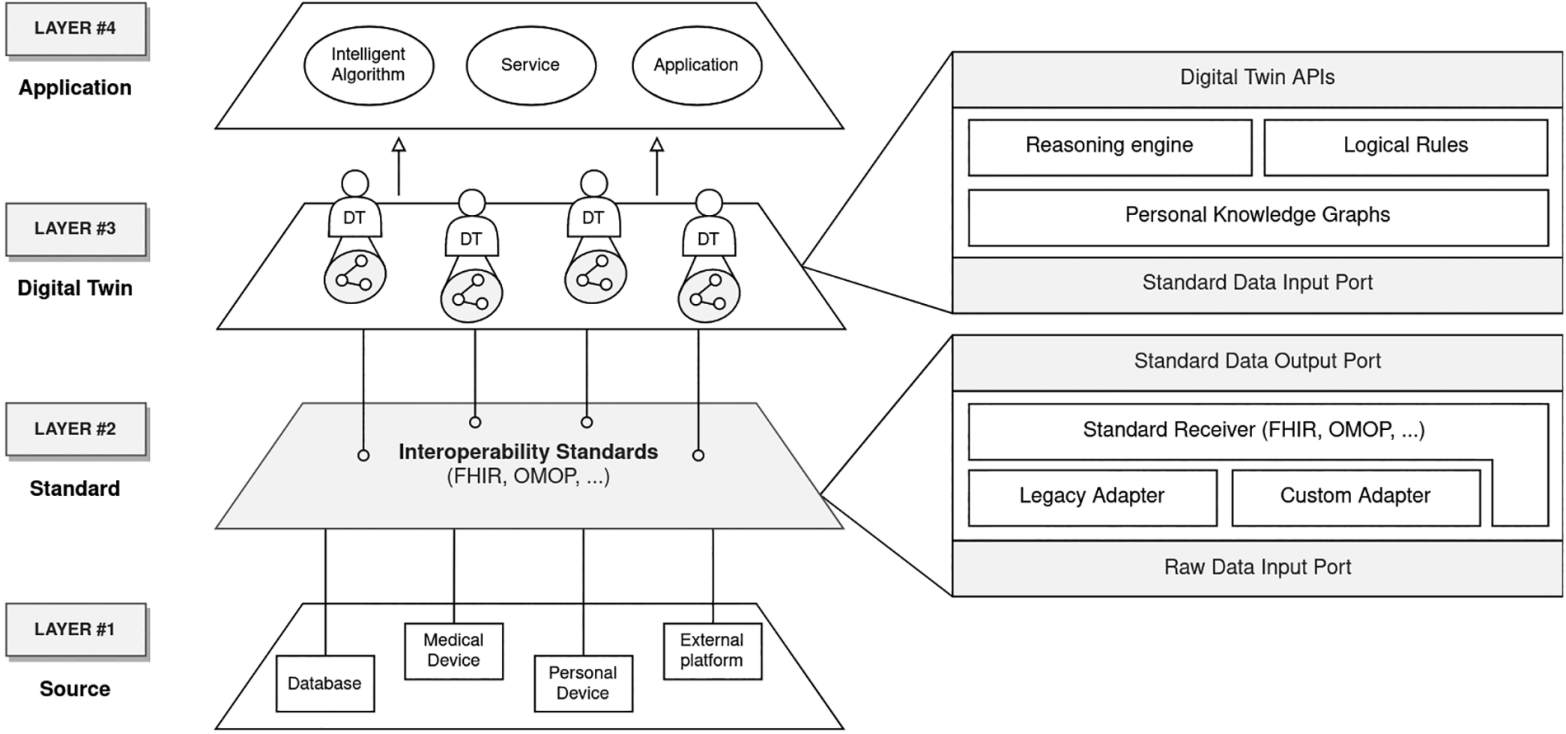
Proposed four-layer architecture. Data flows from the lower levels to the upper ones.

In this section we introduce CONNECTED, a conceptual framework aimed at defining guidelines to develop healthcare applications based on DTs of patients. It has been based on the requirements defined in the previous section, and it has been designed following a modular approach. Hence, the result is a multi-layer architecture in which functionalities are partitioned into horizontal levels (Figure 1): *Source*, *Standard*, *Digital Twin*, and *Application*. The communication between them is monodirectional, so data can flow from one layer to the above one only by passing through designated input and output interfaces.

### 5.1. Level 1: Source

In the healthcare context, a wide array of data sources exists, including personal and medical devices, as well as various platforms and databases. A key aspect of the proposed architecture is its capability to effectively communicate with these sources. Since incoming data may vary in terms of volume, quality, and format, the *Source* layer functions as a sort of funnel. Its primary role is to efficiently manage this diverse information, accommodating different protocols for communication. Ultimately, its aim is to transmit the data stream to the higher-level layer.

### 5.2. Level 2: Standard

The second level of the framework focuses on exploiting standards according to the interoperability requirement defined in Section 4. To this end, the adoption of a well-known solution, such as FHIR,^1^ proves essential in providing domain modeling that is validated by a vast international community of experts. This layer operates as a pipe, in which incoming raw data is harmonized and passed to the *Digital Twin* layer. In this regard, this level must include submodules (*Adapters* in Figure 1) able to process data from legacy standards, and custom or proprietary formats to modern healthcare standards.

### 5.3. Level 3: Digital Twin

To enhance our understanding of patients and their progress, we can utilize all the standardized data so far gathered to populate the DTs. From our perspective, these digital replicas serve as reliable sources of truth, enabling us to track a patient’s evolution over time. While it doesn’t provide simulation capabilities, this DT functions as a versatile repository of data that paints a comprehensive picture of the patient. This data can be leveraged to develop intelligent applications.

Given the heterogeneity of collected information and the constant evolution of the DT model, it is required a data structure capable of managing its changes and expressing semantic relationships between concepts. PKG is identified as an optimal solution to achieve that, as it allows us to capture personal information valuable for a specific subject and integrate it with general concepts of the healthcare domain. This can be done by taking advantage of web semantic tools such as ontologies (e.g., SNOMED-CT) and logical rules (e.g., SWRL). In fact, they can be provided as input to the semantic *Reasoning Engine* aimed at automatically inferring new knowledge about the patient to support a well-informed decision-making process. Finally, DTs offer APIs to run explorative queries on single patients’ PKG. A matching subset of resources is returned to the caller.

### 5.4. Level 4: Application

Since Level 3 doesn’t provide computational resources, the computational models implementing DT applications are located on Level 4. This layer hosts all the applications that need to observe the state of DTs, through their APIs. Following the previously defined requirements, an application is not bound to a single DT, whereas one DT may serve many. Examples of such clients include a wide range of applications, going from simple user interfaces to visualize patient information, to more sophisticated ones like smart assistants to support decision-making or monitoring systems.

## 6. Validation

**Figure 2:**
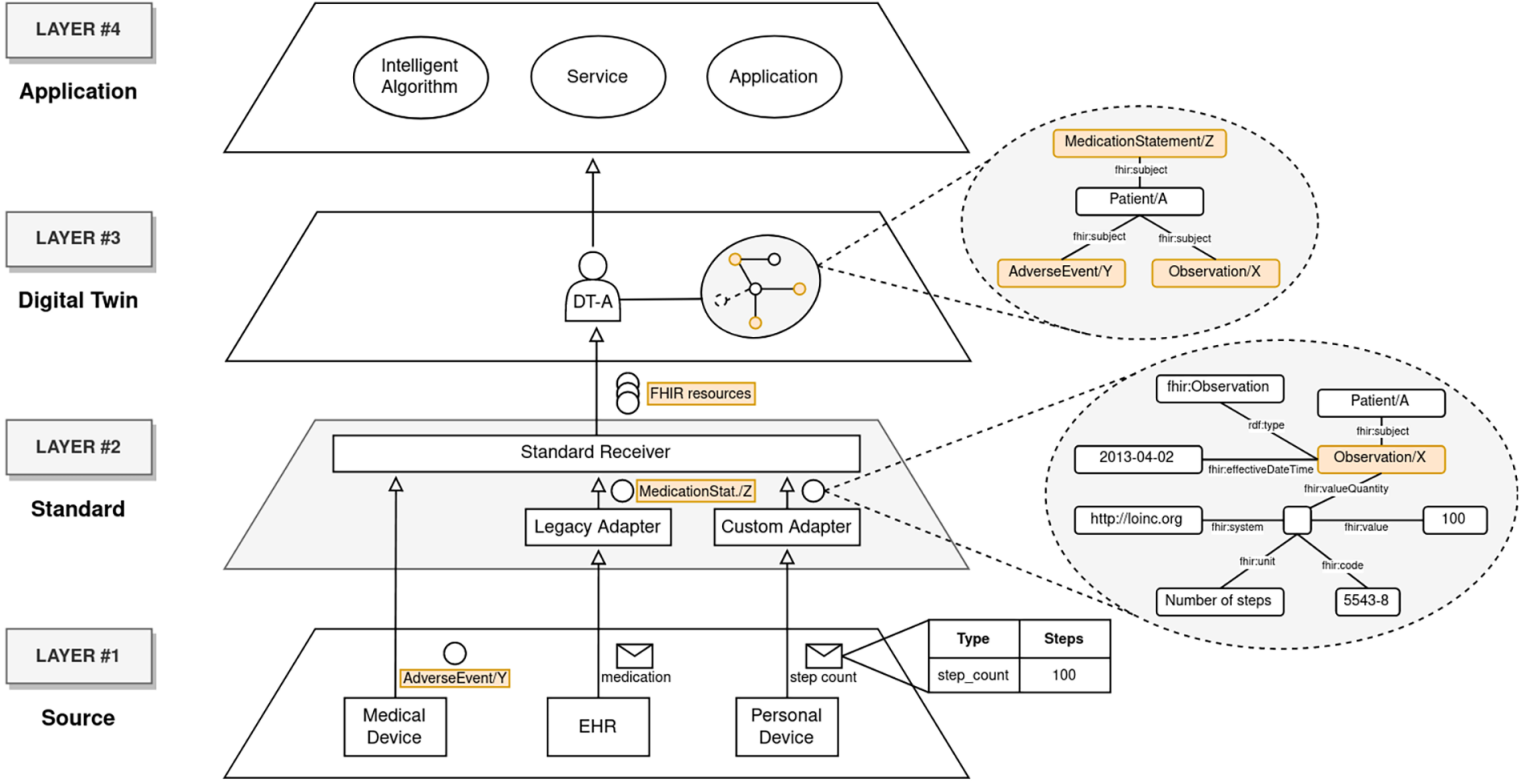
Implementation of the proposed use case through the adoption of CONNECTED.

In this section, we introduce a concrete scenario aimed at clarifying the framework data models and validating its expressiveness. The chosen case study revolves around the identification and prediction of accidental falls in elderly patients, especially those suffering from osteoporosis, a critical domain that benefits from a comprehensive data analysis. This entails capturing heterogeneous information, including patient activity preceding the accident, comprehensive EHR records (e.g. medications, treatments, and previous CT scans), besides measuring the impact of the fall itself. In CONNECTED, these diverse data sources are managed in the *Source* layer, and send messages towards the *Standard* level. Supposing *Medical Device* is FHIR complaint, the fall data is already structured as an AdverseEvent FHIR resource, while data coming from EHR, being based on an HL7 legacy version, requires a conversion step, made by a default adapter (*Legacy Adapter* in Figure 2), transforming it into FHIR resources. As concerns the step count tracked by the patient’s *Personal Device*, it operates using a proprietary format, requiring the development of an ad-hoc adapter (*Custom Adapter* in Figure 2). The resulting information will be finally merged into the Patient DT existing PKG. Leveraging this data will enable smart algorithms to estimate fracture risk for continuous monitoring, facilitating prompt emergency response, if necessary.

## 7. Discussion

In this article, we proposed CONNECTED, a modular framework in which various data sources are integrated through the adoption of modern healthcare standards, to feed general-purpose DTs of patients. Indeed, we identified PKGs as a solid backbone for DTs, given their ability to model heterogeneous data while keeping their high semantic expressiveness and to enable semantic search, supported by a reasoner, which is ideal for complex, dynamic, and rapidly changing environments such as healthcare. General-purpose DTs act as a source of truth and establish a foundation to design and develop smart applications. At last, we presented a use case aimed at validating CONNECTED expressiveness.

Within this perspective, several challenges are yet to be addressed, given the high sensitivity of healthcare data. From a technical point of view, it is fundamental to guarantee the integrity and validity of collected data, ensuring the reliability of the information stored in the PKGs, and fostering their backbone role within the framework. Another primary concern is related to PKG realization. For example, if many PKGs are extracted by a unique underlying graph, it is hard to trace a clear boundary between distinct patients. On the other hand, if PKGs are physically separated, information might be duplicated, possibly leading to inconsistencies. To this end, access-control policies must be implemented to allow specific stakeholders to query only the DTs for which they are given rights. Furthermore, due to the nature of treated information, it is worth mentioning that this kind of platform must deal with complex legal, ethical, and regulatory issues, such as different privacy policies of distinct organizations.

Future work will focus on selecting the best-suited technologies for implementing the proposed framework and its adoption in real-use case healthcare scenarios. In this regard, we are currently developing a solution aimed at unveiling the issues and practical challenges associated with the concrete implementation of the framework. Specifically, we are evaluating the use of Eclipse Hono^2^ for the *Source* layer. This technology empowers the architecture with the ability to communicate with a multitude of IoT devices, regardless of the communication protocol, as well as supporting custom adapters for data transformation. Afterward, standardized data streams are channeled through a distributed and asynchronous message bus, implemented using Apache Kafka^3^. These streams ultimately converge on DTs, where they are stored in a graph database. Our choice for this database is Stardog,^4^ primarily due to its capacity to enable information querying and knowledge inference through the utilization of a reasoner. Our aim is to release the resulting prototype as open-source software for readers to validate them. Finally, further extensions of the framework should be investigated, such as the possibility of integrating computational models into the existing vision of DT, enabling simulation capabilities.

## Data Availability

The original contributions presented in the study are included in the article/supplementary material, further inquiries can be directed to the corresponding author.

## Footnotes

^1^ https://www.hl7.org/fhir/

^2^ https://projects.eclipse.org/projects/iot.hono

^3^ https://kafka.apache.org/

^4^ https://www.stardog.com/
