# Corrigendum: CONNECTED: leveraging digital twins and personal knowledge graphs in healthcare digitalization

**DOI:** 10.3389/fdgth.2024.1416390

**Published:** 2024-05-23

**Authors:** Antonella Carbonaro, Alberto Marfoglia, Filippo Nardini, Sabato Mellone

**Affiliations:** ^1^ Department of Computer Science and Engineering, Università di Bologna, Cesena, Italy; ^2^ Department of Industrial Engineering, Università di Bologna, Bologna, Italy; ^3^ Department of Electrical, Electronic, and Information Engineering “Guglielmo Marconi”, Università di Bologna, Cesena, Italy

**Keywords:** personal knowledge graphs, data integration, healthcare, digital twins, architectural framework

A Corrigendum on CONNECTED: leveraging digital twins and personal knowledge graphs in healthcare digitalization Carbonaro A, Marfoglia A, Nardini F, Mellone S (2023). Front. Digit. Health 5:1322428. doi: 10.3389/fdgth.2023.1322428

## Incorrect funding

In the published article, there was an error in the Funding statement. We used an incorrect CUP number in the following statement “This study was partially supported by the Italian Ministry of University and Research under PNRR-PNC Project PNC0000002 “DARE—Digital Lifelong Prevention” (CUP: B53C22006240001).”

The correct Funding statement appears below.”

## Text correction

In the published article, there was an error. a typographical error was introduced during the review process. As a result, a paragraph appeared erroneously in the article and must be completely removed.

A correction has been made to **Introduction**, paragraph 4, This sentence previously stated:

“First, a review of existing literature about PKGs and DTs is performed. Subsequently, existing architectures that possibly integrate these technologies are analyzed, in order to”

The corrected sentence appears below:

“The authors apologize for this error and state that this does not change the scientific conclusions of the article in any way. The original article has been updated.”

